# Exercise intervention for post-acute COVID-19 syndrome – do FITT-VP principles apply? A case study

**DOI:** 10.17159/2078-516X/2023/v35i1a15284

**Published:** 2023-06-30

**Authors:** G Torres, N Rains, PJ Gradidge, D Constantinou

**Affiliations:** 1 Department of Exercise Science and Sports Medicine, School of Therapeutic Sciences, Faculty of Health Sciences, University of the Witwatersrand, South Africa; 2 N.H.C Health Centre, Northcliff, South Africa

**Keywords:** rehabilitation, recovery, physical therapy, symptom management

## Abstract

The lack of standardisation of reporting exercise interventions hampers the development of best practice guidelines for long COVID patients. This case study on the effect of an exercise intervention in a long COVID patient applied the Consensus on Exercise Reporting Template (CERT) for reporting interventions. FITT-VP exercise prescription principles for long COVID rehabilitation are also suggested. A 58-year-old male, previously hospitalised for 14 days in the ward for the intensive care for the management of severe COVID-19 infection, joined an exercise rehabilitation programme. A medical history, anthropometric, biochemical, lung function, blood pressure, cardiorespiratory fitness and strength measures were all assessed before and after the eight week exercise intervention programme. Positive changes were found in all lung function test measures. Cardiorespiratory fitness, endurance capacity and muscle strength improved. However, the greatest improvements occurred in functional status, fatigue, dyspnoea and the state of depression levels. This case study suggested that in the absence of other instruments, the FITT-VP principles may be used for long COVID patients, and CERT for reporting interventions, but these should be further researched.

Long COVID is a condition characterised by persistent symptoms and/or delayed or long-term complications beyond four weeks from the onset of SARS-CoV-2 infection.^[1]^ A systematic review^[2]^ demonstrated that exercise rehabilitation improves such patients’ symptoms and quality of life. Five randomised control trials (RCTs) included in the review were outpatient/home-based interventions. Only two were considered high quality, indicating the need for further and more robust investigations of the rehabilitation of COVID-19 patients.

The Consensus on Exercise Reporting Template (CERT)^[3]^ was developed to provide a standardised method for the reporting of exercise interventions and lays down a minimum set of 16 key items considered essential to note in replicable exercise programmes. Our case study of these exercise interventions demonstrates the application of the CERT (by describing the 16 essential items). It highlights the need for research in developing FITT-VP exercise prescription principles for long COVID rehabilitation.

## Case report

### History

A 58-year-old male was hospitalised for 14 days in the intensive care ward to manage his severe COVID-19 infection. He had been sedentary before the infection. His disease progression included myocardial involvement, leading to the insertion of a single chamber rate limiting pacemaker on day six of hospitalisation. To regain daily physical functionality, he joined an exercise rehabilitation programme 138 days after discharge. The patient was still symptomatic and reported symptoms of shortness of breath, fatigue, headache, anxiousness, and gastrointestinal upsets. He also reported ambulatory dysfunction (muscle weakness and balance difficulties) and used a walking stick. His prescribed medication was salmeterol xinafoate/fluticasone propionate inhaler (250/25) twice daily.

### Pre-intervention measurements

A medical history and examination were carried out by a sports physician to determine his status and to screen for his entry into the rehabilitation programme. Thereafter a standard protocol was used to collect baseline assessments shown below:

#### Anthropometric measures

Weight (kg) and height (m) were measured using a Seca scale and stadiometer (Model 220, Vogel and Halke, Germany), respectively.

#### Blood pressure

Rested and seated brachial blood pressure (mmHg) with an average of two were recorded on the dominant arm using an automated blood pressure cuff (Fora Active Plus P30, FaraCare Suisse, Switzerland).

#### Pulse oximetry

Pulse oximeter readings were measured in a rested, seated position using the Berry Pulse Oximeter (BM1000E, Shanghai Berry Electronic Tech Co., Ltd).

#### Biochemical analysis

Venous blood samples were collected and analysed at a commercial pathology laboratory for C-Reactive Protein (CRP) (inflammatory marker) and Interleukin 6 (IL-6) (immune activation marker).

#### Lung function test

Flow volume loop spirometry was performed using a standardised procedure with the computer-based Koko PFT spirometry (Koko PFT Ltd, Waltham Abbey, Essex, UK).

#### Cardiorespiratory fitness (CRF)

A submaximal CRF test was performed using a cycle ergometer (Wattbike, Nothingham, England) (Ramp Test mode). Resting and effort electrocardiograms were recorded (QRS Universal ECG, San Diego, USA) and capillary blood lactate concentration was measured (Accutrend Plus, Roche, Mannheim, Germany). The initial workload was 25W, with increments of 20W every three minutes, until functional volitional fatigue was reached. During the test, ratings of perceived exertion, blood lactate, blood pressure and heart rate measures were collected (the last 30 seconds of each workload). VO_2_ peak was predicted using equations from the FRIEND registry.^[4]^

#### Hand grip dynamometer strength test

Muscle strength was measured using a hand grip dynamometer (Takei Kiki Kogyo, Japan). The best of three trials were recorded for both hands.

#### Questionnaires

The Post-COVID-19 functional status scale^[5]^ was administered to assess daily functionality. The Fatigue Assessment Scale was used for fatigue levels and the MRC Dyspnea Scale was used to assess breathlessness and difficulty in breathing. The Hamilton Depression Rating Scale (HAM-D) was used to assess levels of depression.

### Exercise intervention

The exercise intervention is described using items from the Consensus on Exercise Reporting Template (CERT).^[3]^

#### 1. Detailed description of the type of exercise equipment

A treadmill and stationary exercise bike were used for endurance training. Dumbbells, Pilates balls, elastic bands, and strength equipment which controlled movement (e.g. leg press machine and pectoral deck machine) were used for resistance training.

#### 2. Detailed description of the qualifications, expertise and/or training

An exercise physiologist (PhD) and two assistants (Honours in Biokinetics) delivered the exercise intervention. A standard operating procedure document was developed and used for the intervention.

#### 3+4. Describe whether exercises were performed individually or in a group; supervised or unsupervised; how they were delivered

All exercise sessions were supervised, performed individually and delivered face-to-face, monitored and tracked via the Technogym 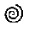 (TG) MyWellness mobile application.

#### 5. Detailed description of how adherence to exercise was measured and reported

All exercise sessions were verified by direct supervision or by heart rate monitoring via Polar monitor and captured on the MyWellness 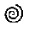 mobile app. Blood pressure, heart rate, RPE, O_2_ stats and symptoms were monitored by the supervising exercise professional during all exercise sessions. Data were recorded on a spreadsheet. Adherence was calculated as a percentage, equal to an actual number of exercise minutes completed each week at the appropriate intensity, divided by the total number of minutes prescribed. The adherence for supervised sessions was 85%.

#### 6. Detailed description of motivation strategies

Verbal encouragement and positive reinforcement were applied during sessions. The Self-determination theory of behaviour change and the Health Belief Model that highlight self-efficacy, individual autonomy, perceived benefits and the affective value of a goal/action were used as motivation strategies. Links between effect (feelings, emotions) and a particular physical activity/movement were highlighted during exercise.

#### 7a+b. A detailed description of the decision rule(s) for determining exercise progression and how the exercise programme progressed

Exercise progression was informed by and followed the flowchart set out in the British Medical Journal.^[6]^ Progression included small increments based on patient feedback and responses to exercise.

#### 8. Detailed description of each exercise to enable replication

The exercises were all made available as videos on the TG MyWellness 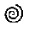 mobile application and accessible by a website link (www.technogym.com/za/technogymapp-za/).

#### 9. Detailed description of any home programme component

In week five, the participant was confident enough to add walking one-two times a week, of 10–20 min each, outside of supervised sessions.

#### 10. Describe whether there are any non-exercise components

No other components were added to the exercise intervention. However, it was noted that a psychological component was needed and would have been beneficial to help the high levels of participant anxiety.

#### 11. Describe the type and number of adverse events that occur during exercise

Dizziness during some exercise sessions was experienced by the participant in the first four weeks but always subsided quickly (four events). Sessions were adjusted for dizzy spells.

#### 12. Describe the setting in which the exercises are performed

All exercise sessions were performed at the Medical Exercise facility, Department of Exercise Science and Sports Medicine, University of the Witwatersrand, South Africa.

#### 13. Detailed description of the exercise intervention

See Supplement 1: Table 3.

#### 14a+b. Describe whether the exercises are generic (one size fits all) or tailored and how exercises are tailored to the individual

Exercises were based on current known exercise prescriptions for patients presenting with long COVID and tailored to the individual based on heart rate response, RPE, dyspnoea level, exercise technique and symptoms.

The exercise programme focussed on large muscle group exercises, with a controlled plane of movement (strength equipment), as for a sedentary or beginner resistance training programme. The specific exercises chosen (e.g. bench press machine) were based on available equipment. Exercises were chosen to activate similar muscle groups and movement patterns. For example, when free-standing squats caused knee pain, they were replaced with wall squats using a Pilates ball.

#### 15. Describe the decision rule for determining the starting level

The heart rate at the onset of blood lactate accumulation measured during the CRF test determined the starting level for endurance exercise. The starting level for the resistance exercises was based on exercise technique.

#### 16a+b. Describe how adherence or fidelity is assessed/ measured and the extent to which the intervention was delivered as planned

See item 5.

### Results and outcomes

Data were analysed to compare baseline and post-intervention outcome measures (Tables 1 and 2).

All the lung function test variables showed positive changes. The improvements in cardiorespiratory fitness and endurance capacity are clearly shown by the changes in VO_2_ peak and blood lactate threshold. Muscle strength and blood inflammatory marker also improved (Tables 1 and 2). However, the greatest improvements occurred in functional status, fatigue, dyspnoea and the state of depression levels. Body weight and blood pressure measures showed negative changes (latter within normal reference ranges).

## Discussion

The greatest effects of exercise intervention in this case study were in cardiorespiratory fitness, self-reported functional status, fatigue, dyspnoea and the state of depression levels. The unexpected increases in resting heart rate and resting systolic and diastolic blood pressures in the sessions may be attributed to autonomic nervous system effects, e.g. a stressful drive on post-intervention testing day compared to pre-intervention testing day. Another possible explanation for the result is that COVID infection could still be lingering in the autonomic nervous system.^[7]^

The improvement in the blood inflammatory marker may have been a combined result of the healing process after hospital discharge (i.e. the participant started to exercise 138 days after hospital discharge) and the effect of the exercise programme.

FITT-VP principles, specifically for long COVID rehabilitation, have not been developed. After using these techniques in various exercise scenarios, we decided to implement the adjustable approach for long COVID since there were no other guidelines available. The following FITT-VP principles are suggested based on the outcomes of the exercise intervention of this case study:

### Frequency

2 supervised sessions and 1 unsupervised, walking session per week.

### Intensity

65–75 % HR_peak_, RPE: 6–11

### Time

7–10 min endurance exercises + 1–4 resistance exercises: 1 set of 4–8 reps

**Walking (Home):** 6–10 minutes at RPE of 6–11

### Type

**Endurance exercises:** (treadmill, walking or cycle ergometry)

**Resistance exercises:** (including balance) using strength equipment, dumbbells, wellness balls, bands, body weight+ flexibility (2–4 per session)

### Volume

Cannot be set, needs to be individualised – based on responses of the individual

### Progression

**Endurance exercises:** Start at 7 minutes, add 1–2 min every week; after 3 weeks increase %HR peak by 5%

**RPE**: Week 1–4: 6–9; Week 5–8: 9–11.

**Resistance exercises:** Start:1 set of 4–8 reps, increase by 2 reps every week and when reached 10 reps, add 1 set every week until 3 sets of 10 reps; + add 1 exercise every 2 weeks

Further, to standardise reporting in interventions, the CERT is suggested. However, these suggestions need to be researched with further explorative study designs, to recommend detailed exercise prescription for long COVID rehabilitation.

Importantly, long COVID symptoms vary vastly per individual, as do responses and adaptations to exercise intervention. Thus, the principles need to be flexible and adjusted accordingly to the varied patient responses that may be observed/experienced.

## Conclusion

A combined endurance and resistance exercise programme had positive effects on cardiorespiratory fitness, functional status, fatigue and depression levels, with no deleterious effects in parameters that did not show improvement. Based on this case study, we recommend using FITT-VP principles as a helpful solution for long COVID patients until more advanced methods are discovered through additional research. It is also suggested to adopt standardised reporting using CERT.

## Supplementary Information



## Figures and Tables

**Table 1:** Changes in anthropometric, cardiorespiratory and blood parameters after eight weeks of exercise intervention

Parameter	Pre−	Post−	% change	Absolute change

Physical activity level (kcal.wk-1)	Unknown	763		
Weight (kg)	129	131	+1.4	+1.8
BMI (kg.m-2)	35.0	35.5	+1.4	+0.5
Resting heart rate (b.min-1)	73	79	+8.2	+6
Resting oxygen saturation level (%)	95	95		
Systolic blood pressure (mmHg)	120	133	+10.8	+13
Diastolic blood pressure (mmHg)	81	89	+9.8	+8
Resting ECG	Normal	Normal	-	-
FVC (L.min-1)	3.70	4.02	+8.6	+0.32
FEV_1_/FVC (%)	71	78	+9.8	+7
PEFR (L.s-1)	6.23	6.95	+11.6	+0.72
Blood CRP level (mg.l-1)	25	3	−88	−22
Blood IL-6 level (pg.ml^−1^)	1.8	-	-	-

BMI, body mass index; ECG, electrocardiograms; FVC, forced vital capacity; FEV_1_; forced expiratory volume in one second; PEFR, peak expiratory flow rate; CRP, C-Reactive Protein; IL-6, Interleukin 6.

**Table 2:** Changes in cardiorespiratory (CRF) test, strength and qualitative scale parameters, after eight weeks of exercise intervention

Parameter	Pre−	Post−	% change	Absolute change

Predicted VO_2_ peak (ml.kg^−1^.min^−1^)	8.9	13.8	+ 55.1	+4.9
Power at onset of blood lactate accumulation (W)	53	64	+ 20	+10.6
Maximal power /Mass (W.kg^−1^)	0.67	0.80	+ 19.4	+0.13
O2 saturation before CRF test (%)	95	94	−1.05	−1
O2 saturation after CRF test (%)	94	94		
Hand grip strength (kg)	42	44	+4.8	+2
Post COVID-19 functional status scale	3*	2*	−33.3	−1
Fatigue assessment scale	27 (fatigue)	17 (normal)	−37	−10
Medical research council dyspnea scale	4**	3**	−25	−1
The Hamilton Depression Rating Scale (HAM-D)	26 (very severe depression)	16 (moderate depression)	−38.5	−10

* A lower number means a better functional status;

** A lower number denotes less dyspnoea. CRF test data are provided in the supplementary Table 4.
