# Supplementary material for: Exercise intervention for post-acute COVID-19 syndrome – do FITT-VP principles apply? A case study
Source: S Afr J Sports Med. 2023 Jun 30;35(1):v35i1a15284. doi: 10.17159/2078-516X/2023/v35i1a15284 (PMC10798601; doi:10.17159/2078-516X/2023/v35i1a15284)
Supplement: Supplementary file 1 [file 2078-516X-35-v35i1a15284-s001.pdf]

Exercise intervention for post-acute COVID-19 syndrome – do FITT-VP principles apply? A case study

Supplementary Tables

Table 3. Detailed description of exercise intervention

Exercise	Week 1	Week 2	Week 3	Week 4	Week 5	Week 6	Week 7	Week 8
**Endurance**								
Walking treadmill (min)			10	12	12	14	16	18
Cycle ergometer (min)	7	9						
Intensity %HR_{peak}	60%	60%	60%	65%	70%	70%	75%	75%
RPE scale number	6	6	7	7	8	9	10	11
**Resistance**								
Wall squat	1 x6	1 x8	1 x10	2 x10	3 x10	3 x12	3 x12	3 x12
Machine bench press		1 x6	1 x8	1 x10	2 x10	3 x10	3 x12	3 x12
Lunges				1 x6	1 x8	1 x10	2 x10	3 x10
Machine low row pull						1 x6	1 x8	1 x10
Machine adductor							1 x6	1 x8
Machine abductor							1 x6	1 x8
**Neuro-muscular**								
Standing on one leg (sec)		1 x10	1 x10	1 x15	1 x20	1 x20	1 x25	1 x30
Calf raises on stability mat (sec)				1 x10	1 x15	1 x20	1 x20	1 x25
Abdominal prep exercise			1 x10	1 x12	1 x12	2 x10	2 x12	3 x10
**Flexibility**	2-4 exercises per session<br>Supine leg raise, hamstring stretch<br>Supine, 90° hip flexion and side rotation stretch<br>Triceps, behind the head, stretch<br>Standing calf stretch<br>Deltoid, arm across the chest, stretch							

Table 4. Cardiorespiratory fitness (CRF) test data

Stage time (min)	Workload (Watts)	Heart rate (bt/min)		Blood pressure (mmHg)		RPE		Dyspnea	
		Pre	Post	Pre	Post	Pre	Post	Pre	Post
**REST**		73	79	120/81	133/89				
**0-3**	25	87	103			9	8	0	0
**3-6**	45	97	110			10	8	1	1
**6-9**	65	110	114			13	9	4	1.5
**9-12**	85		122				11		2
**12-15**	105		128				13		3
**15-16 (stopped after 1 min)**	125		135				15		3.5
**Immediately post-ex**		97	111	143/89	153/93				
